# Predicting Road Conditions with Internet Search

**DOI:** 10.1371/journal.pone.0162080

**Published:** 2016-08-29

**Authors:** Nikolaos Askitas

**Affiliations:** IZA - Institute for the Study of Labor, Bonn, Germany; Georgia Institute of Technology, UNITED STATES

## Abstract

Traffic congestion is an important problem both on an individual and on a societal level and much research has been done to explain and prevent their emergence. There are currently many systems which provide a reasonably good picture of actual road traffic by employing either fixed measurement points on highways or so called “floating car data” i.e. by using velocity and location data from roaming, networked, GPS enabled members of traffic. Some of these systems also offer forecasting of road conditions based on such historical data. To my knowledge there is as yet no system which offers advance notice on road conditions based on a signal which is guaranteed to occur in advance of these conditions and this is the novelty of this paper. Google Search intensity for the German word stau (i.e. traffic jam) peaks 2 hours ahead of the number of traffic jam reports as reported by the ADAC, a well known German automobile club and the largest of its kind in Europe. This is true both in the morning (7 am to 9 am) and in the evening (4 pm to 6 pm). The main result of this paper is then that after controlling for time-of-day and day-of-week effects we can still explain a significant additional portion of the variation of the number of traffic jam reports with Google Trends and we can thus explain well over 80% of the variation of road conditions using Google search activity. A one percent increase in Google stau searches implies a .4 percent increase of traffic jams. Our paper is a proof of concept that aggregate, timely delivered behavioural data can help fine tune modern societies and prompts for more research with better, more disaggregated data in order to also achieve practical solutions.

## Introduction

According to the German automobile club ADAC, in 2014 there have been 475,000 traffic jams on German highways which amounted to 960,000 kilometres of jammed traffic. These numbers represent an increase of 14.4% and 15.6% respectively compared to the year before. This is just an instance of a multiyear trend which is only expected to get worse even though the report of the ADAC estimates that most of the current increases are due to progress in the method of documentation of traffic jams. According to the ADAC report (https://www.adac.de/infotestrat/adac-im-einsatz/motorwelt/Staubilanz2014.aspx) these traffic jams amounted to 285,000 lost hours which is 32 years.

Adverse road conditions such as traffic congestion are known to cause a host of undesired side effects (see for example [1]). These vary from increased pollution (e.g. gas emissions), energy waste, additional transportation and production costs to waste of labor and delays in product deliveries. Traffic congestion is also known to impact public health negatively ([2]), to cause stress and road rage ([3]) and to even affect the unborn ([4]). If we thought of a city, a region, a country or any other social unit as a large living organism road traffic would be one of its circadian rhythms and traffic jams would be an obstruction to its entrainment. It is hence not surprising that obstructing the smooth flow of traffic sends ripples of negative effects deep into many aspects of socioeconomic life. Understanding, forecasting and preventing adverse road conditions is therefore important for the benefit of the drivers but also obviously for economic reasons.

The emergence of traffic jams is a complex matter and may have many causes. Some are simple and have to do with fluctuating degradation of the available infrastructure (e.g. accidents, road constructions, weather and the like) and some are more complex and depend on behavioural, topological and dynamical complexities. Two well known such complex phenomena are discussed in [5], where it is shown that expanding the road network may paradoxically worsen road performance and in [6] where the spontaneous emergence of the so called “phantom jams” are experimentally investigated or in [7] where a theoretical model is discussed. There is an extensive literature both empirical and theoretical which develops “fundamental diagrams of traffic” i.e. studies the relationships between traffic density, velocity and flow (see e.g. [8] and the literature therein). The basic intuition in this type of research is that the higher the traffic density is the more likely it becomes that driver actions are interlinked: high density implies more or less that if one driver breaks then all drivers do so as well with an increasing abruptness. Once a critical density is reached traffic will almost certainly stall. Forecasting and preventing traffic jams is hence a hard problem.

If we had a global ledger where future traffic participants would volunteer their itineraries in advance things might be different. We could then get an advance estimate of future traffic and even try to prevent traffic density from reaching critical mass by deploying a kind of leaky bucket algorithm (see [9]) by which we queue departures and still get drivers to their destinations faster than otherwise. Of course such a ledger of itineraries is highly unrealistic but Google search data might be a good proxy.

What an individuals searches for in Google reveals something about their state or their intent. If they search for a recipe we know what they might cook, if they search for the bus departure timetable they might take the bus and if they search for information on traffic congestion along a certain highway they are likely to drive on it at a later point in time. The core observation which sparked this paper is that every morning stau searches in Germany peak at 7 am and then they start to dissipate as drivers are being injected into the traffic. Two hours later, at 9:00 am, we have the morning peak of ADAC traffic reports. Similarly at 16:00 hrs every afternoon we observe the search peak and two hours later, at 18:00 hrs, we see a peak of the ADAC traffic reports. Clearly performing a Google search and driving at the same time are mutually exclusive and this is what creates this advance. A future proliferation of mobile applications which allow one to speak searches into a cell phone as well as to graphically get current crowdsourced traffic conditions may diminish the effectiveness of this method but currently such search intensity is a good proxy for the number of people who intent to drive soon. By searching for traffic jam information ahead of driving, drivers provide us with advance warning of their intent to drive.

The number of people who will drive every morning has some regularities, like 24-hour or 7-day periodicities, but it also has a certain stochastic component. The latter cannot be forecasted with historical data as it is impossible to predict when some people will be redeployed by their work or have a private emergency and hence change their driving plans. In this paper I propose a formula for estimating the country wide, aggregate number of hourly ADAC traffic jam reports which has time of the day, day of the week and hourly stau search intensity from two hours ago as its explanatory variables. I hence can forecast this crude indicator of road conditions two hours in advance. There are currently several systems which provide live road conditions. They are so called “probe-based” systems or based on fixed measurement points on highways and may use crowdsourcing of volunteers, leveraging the fact that they are roaming members of a digital network and are equipped with Global Positioning Systems able to compute and transmit position, direction and velocity of motion. Such systems include Google Traffic, a feature of Google Maps and the crowdsourcing system of INRIX. More providers can be found here: https://en.wikipedia.org/wiki/Traffic_reporting. Lastly the literature cited in [10] covers a good portion of the literature which uses geo-located data to study mobility questions.

To my knowledge there is no system available which can have as much as two hours advance on the emergence of such road conditions and does so not by using an algorithm trained on past values of road conditions but by using behavioural measurements guaranteed to occur before the emergence of road congestion. Such current “floating car data” systems (e.g. [11]) can predict at most 15-30 minutes ahead. My target variable is a crude proxy for road conditions but this paper is a proof of concept that with better target data Google Search can be a powerful predictive tool of traffic conditions. In fact this paper is among very few in the literature of Google Search data which uses hourly data, has a clear behavioural foundation and detects two phenomena with a causal phase difference. Moreover it suggests that the Google Traffic team ought to work closer with the Google Trends team to better forecast traffic. Two hours advance notice is an enormous amount of time when it comes to traffic jams.

## Materials and Methods

### ADAC traffic jam data

**Fig 1:**
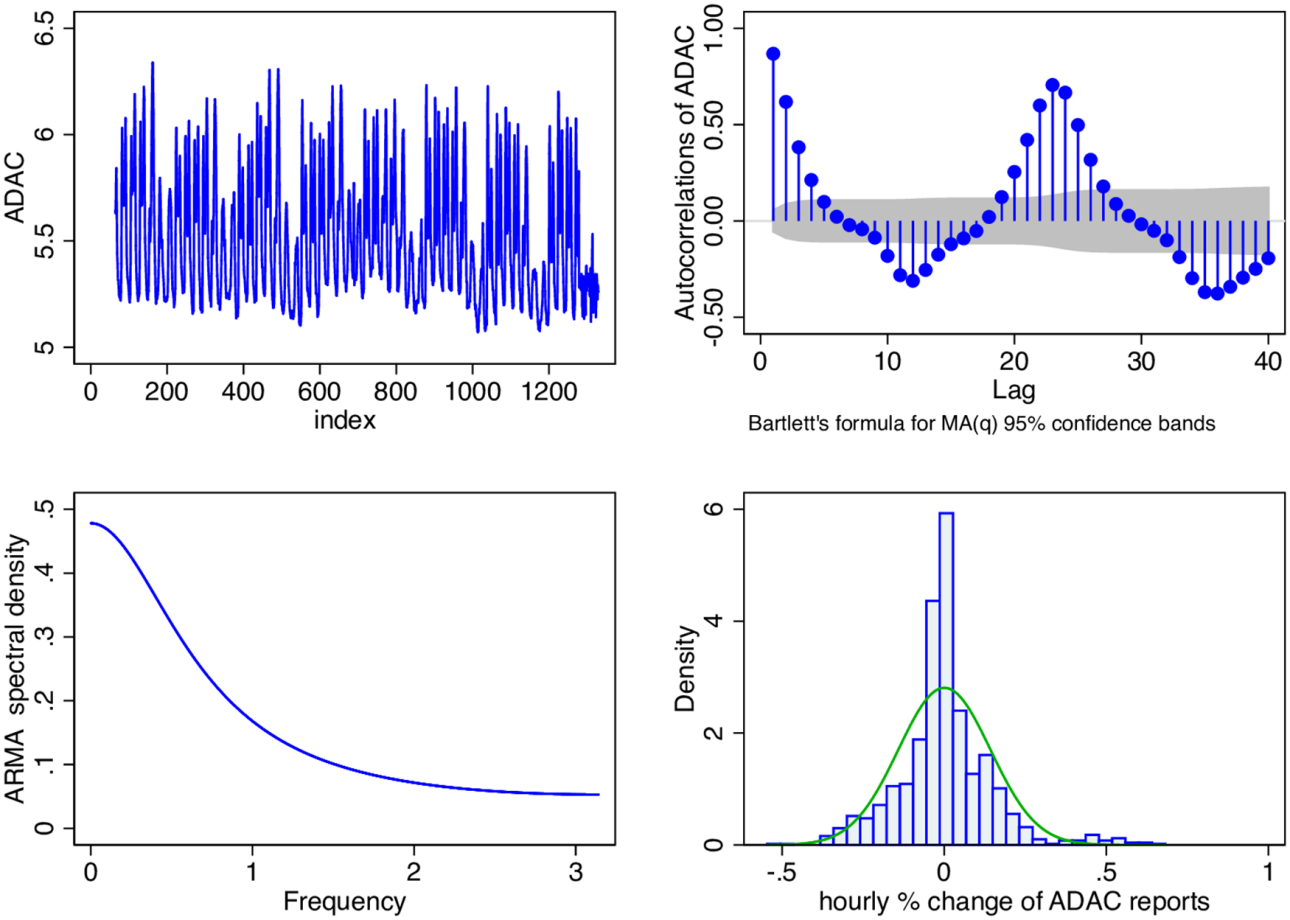
ADAC traffic jams. Natural logs of hourly ADAC traffic jam reports (top left), its autocorellations (top right), spectral density (bottom left) and histogram of percentage changes (bottom right). Data Source: ADAC (adac.de) and own calculations.

On September 28 at 14:59 hrs I started collecting ADAC traffic jam data, programmatically every five minutes, from the website (https://www.adac.de/reise_freizeit/verkehr/aktuelle_verkehrslage/default.aspx) of the German automobile club ADAC. Prior to resorting to data scraping I had contacted ADAC about access to their past data but unfortunately I could not elicit a response. The data collection for the purpose of this paper ends on November 14 2015 at 13:59 hrs. Each observation consists of a timestamp, region name and current count. I use the countrywide aggregate, average hourly count as my target time series. Fig 1 depicts some diagnostics for this data.

As expected the series exhibits a significant amount of autocorellation, a strong circadian rhythm with day-of-week and hour-of-day fixed effects and, due to non-linearities and endogeneities, a leptokurtic and fat tailed, unimodal distribution of hourly percentage changes. The mean hourly number of traffic jams in that period is 177 with a standard deviation of 80, a minimum of 80 and a maximum of 486. The data on the numbers of traffic jams on the ADAC website is based on reports from volunteers who call in to report the traffic congestion they are currently in. In that respect it is the “low tech” version of, so called, probe-based traffic reporting which use floating car data, whose more modern high tech variant is done with roaming, networked GPS capable smartphones. This means that our target variable could very well have been the data from systems such as Google Traffic, INRIX or any other system which uses floating car data to reflect current road conditions. The fact that we use the ADAC data is simply a result of data availability.

### Google Trends Data

**Fig 2:**
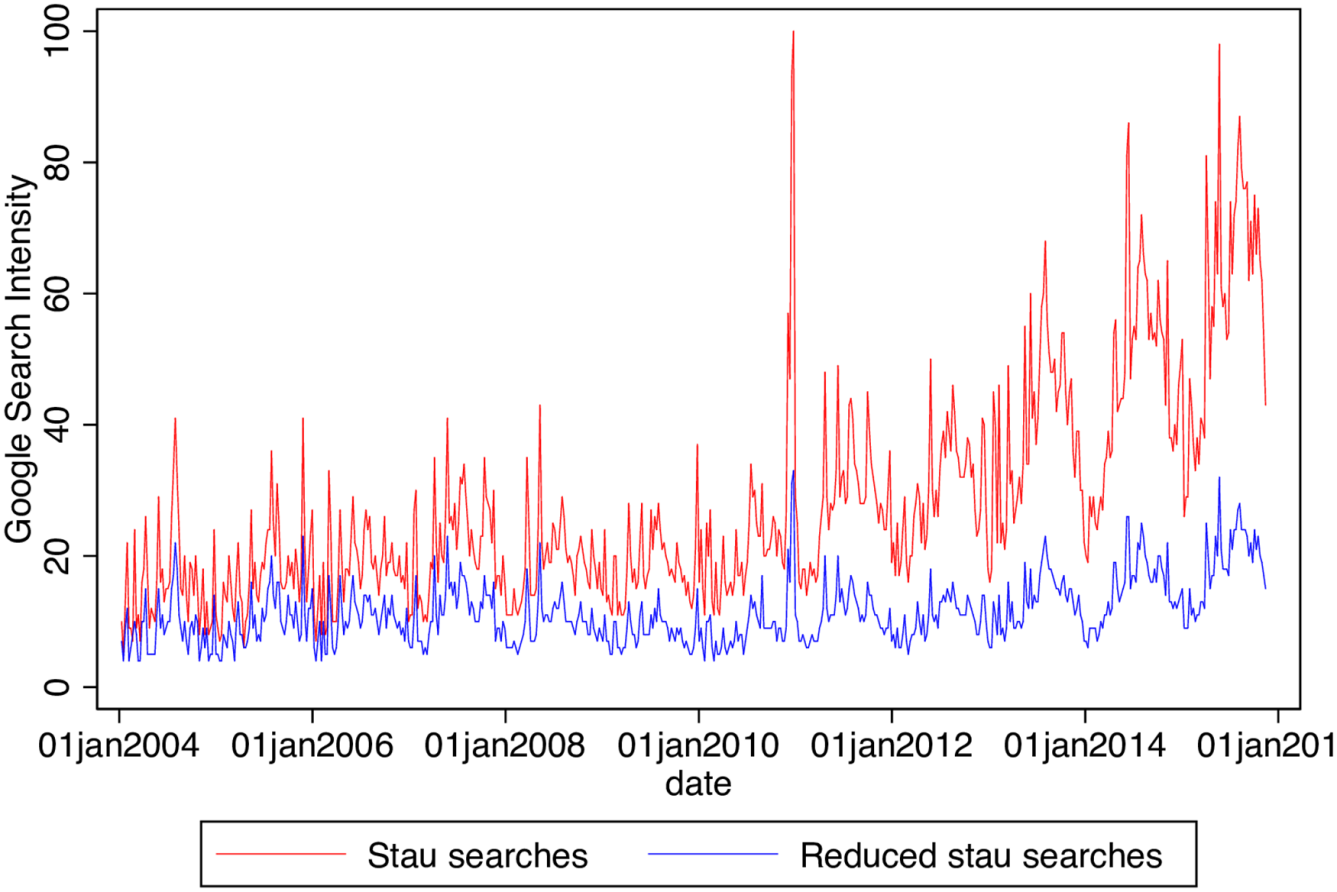
Google Searches for “stau.” The reduced series are searches containing the word stau without containing any of: nrw, a7, a2, a3, a1, wdr, a8, a5, aktuell, autobahn, a9, info, swr3, bayern, hamburg, a4, staumelder, adac, berlin, a6, verkehr, ffh, hessen, köln, münchen, swr, a81, a61, deutschland. A 60% of the total stau search volume is accounted for by these words. Data Source: Google Trends (www.google.com/trends) and own calculations.

The Google Trends data are hourly data of searches containing the word stau which is German for traffic jam. They are obtained from the Google Trends data provisioning system which can be found by pointing one’s browser at the Google Trends website (https://www.google.com/trends/). I am discussing some aspects of Google Trends data in [12]. The reader who wants to use this data may also want to consult a more practical data description in [13]. Before moving on to discussing the hourly data I dissect such searches in order to provide support for my identification strategy. In this way I demonstrate that assuming that the searches are made by drivers is not entirely unfounded. Fig 2 shows weekly searches which contain the word “stau” since 2004 together with a reduced series. The enormous spike on December 2010 is due to an extraordinary traffic jam in Germany due to snowfall. The trend in this figure is in agreement with the annual ADAC traffic jam reports.

The reduced series is constructed by subtracting those searches which contain any of the top 30 additional words. We see that these 30 words account for 60% of the total volume on the average. These keywords are city or region names (NRW, Bayern, Hamburg, Berlin Hessen, Köln, München, Deutschland) or names of highways (A7, A2, A3, A1, A8, A5, A9, A4, A6, A81, A61). Such searches even reveal which highway the driver will be driving on. We also have radio stations (WDR, FFH, SWR) and other websites such as the one of ADAC. Although we cannot subtract them from the volume due to limitations from the Google Trends data provisioning system we know a further set of keywords which are contained in the reduced series. These keywords further support the thesis that these are prospective drivers. They are: a45, stuttgart, a40, elbtunnel, frankfurt, a44 stau, meldungen, hr3, a14, app, hannover, online, a24, niedersachsen, ndr, bremen, a46, a10, staumeldungen, aktuelle, karlsruhe, wdr2, a31, a57, a43, baden württemberg, nürnberg, bw, nachrichten, antenne, dresden, b10 stau, a7, a96, österreich, sachsen, bonn,a5. The highway numbers contained as well as the city and region names imply that our method can predict not just the nationwide aggregate road conditions but also regional conditions. The largest portion of stau searches are those that also contain the string “NRW” which stands for Nord-Rhein-Westfalen the region with the densest highway network in Germany as can be seen by consulting www.autobahnatlas-online.de.

Table 1 depicts the towns from where searches for four major highways originate.

**Table 1:** Origins of four highway-specific Google “stau” searches.

stau A1	stau A2	stau A3	stau A7
Glauchau	Glauchau	Glauchau	Glauchau
Bremen	Gütersloh	Oberhausen	Flensburg
Oldenburg	Bielefeld	Düsseldorf	Drochtersen
Vechta	Hanover	Cologne	Norderstedt
Osnabrück	Brunswick	Koblenz	Hamburg
Münster	Wolfenbüttel	Aschaffenburg	Hanover
Dortmund	Magdeburg	Würzburg	Göttingen
		Erlangen	Kassel

For the highways A1, A2, A3, A7 searches for stau A1, stau A2, stau A3 and stau A7 come from towns along the respective highways.

The reader may easily verify that the towns where these searches originate from are situated along the respective highways thus providing support for the claim that such data can be used for regional traffic forecasting as they can be thought of as early itinerary announcements of sorts. An interesting accidental observation is striking from the table. The town Glauchau appears in the top position for all highways. In other words this town in an exception to the rule of searches for a highway coming from along the highway. This leads to the conjecture that something beyond and above end user behaviour happens there. Very close to the town is Volkswagenwerk Zwickau the well known automaker. It would appear as though this automaker may already be looking into this type of data.

**Fig 3:**
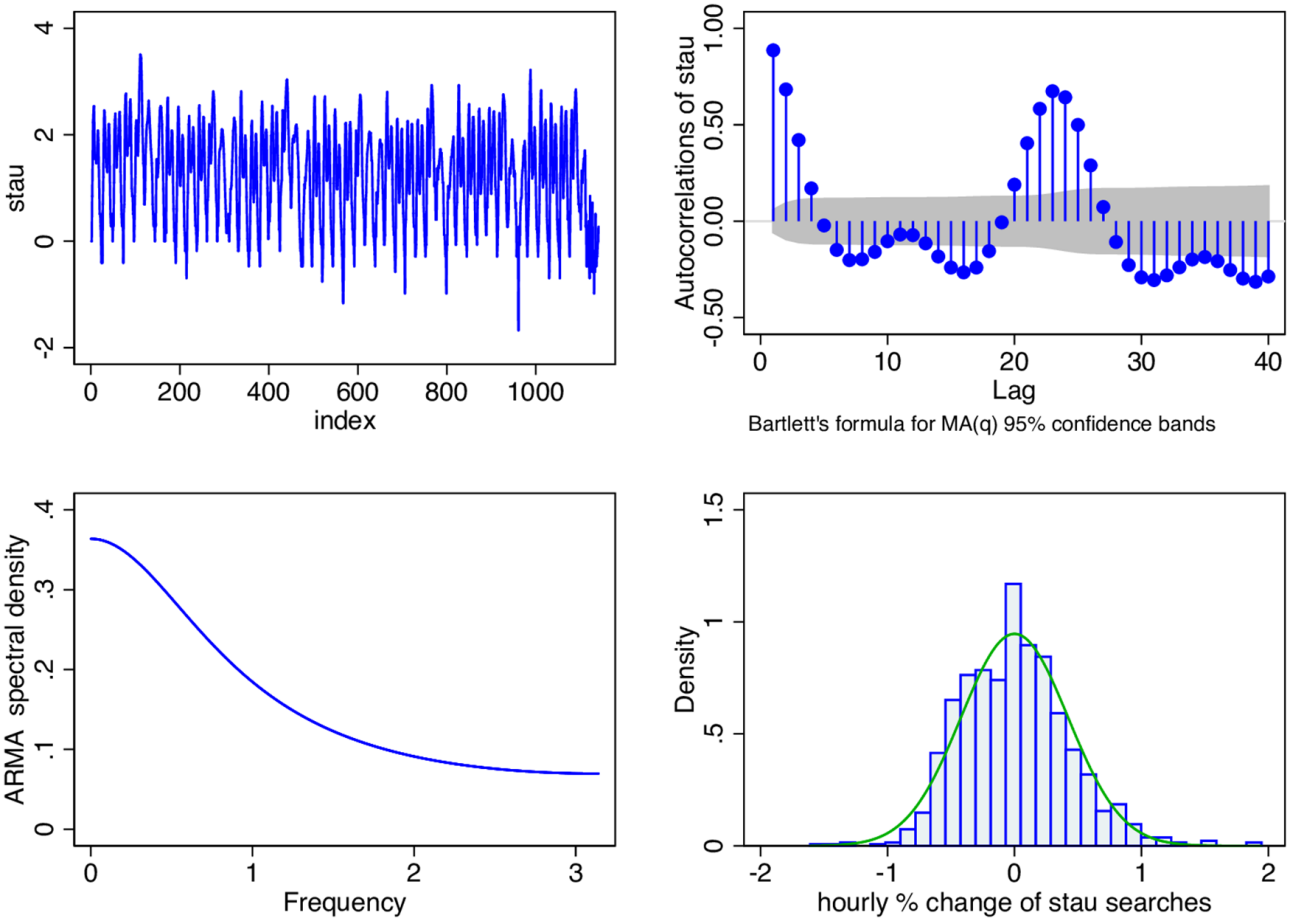
Hourly Google Searches for “stau.” The natural logs of the hourly stau search intensity (top left), its autocorellations (top right), spectral density (bottom left) and histogram of percentage changes (bottom right). Data Source: Google Trends (www.google.com/trends) and own calculations.

To recap, by looking at Google stau searches not only do we hope to parsimoniously identify soon-to-be members of traffic but furthermore to locate the highway they will soon be on. The characteristics of the hourly series of Google searches for stau can be seen in Fig 3.

## Results

**Fig 4:**
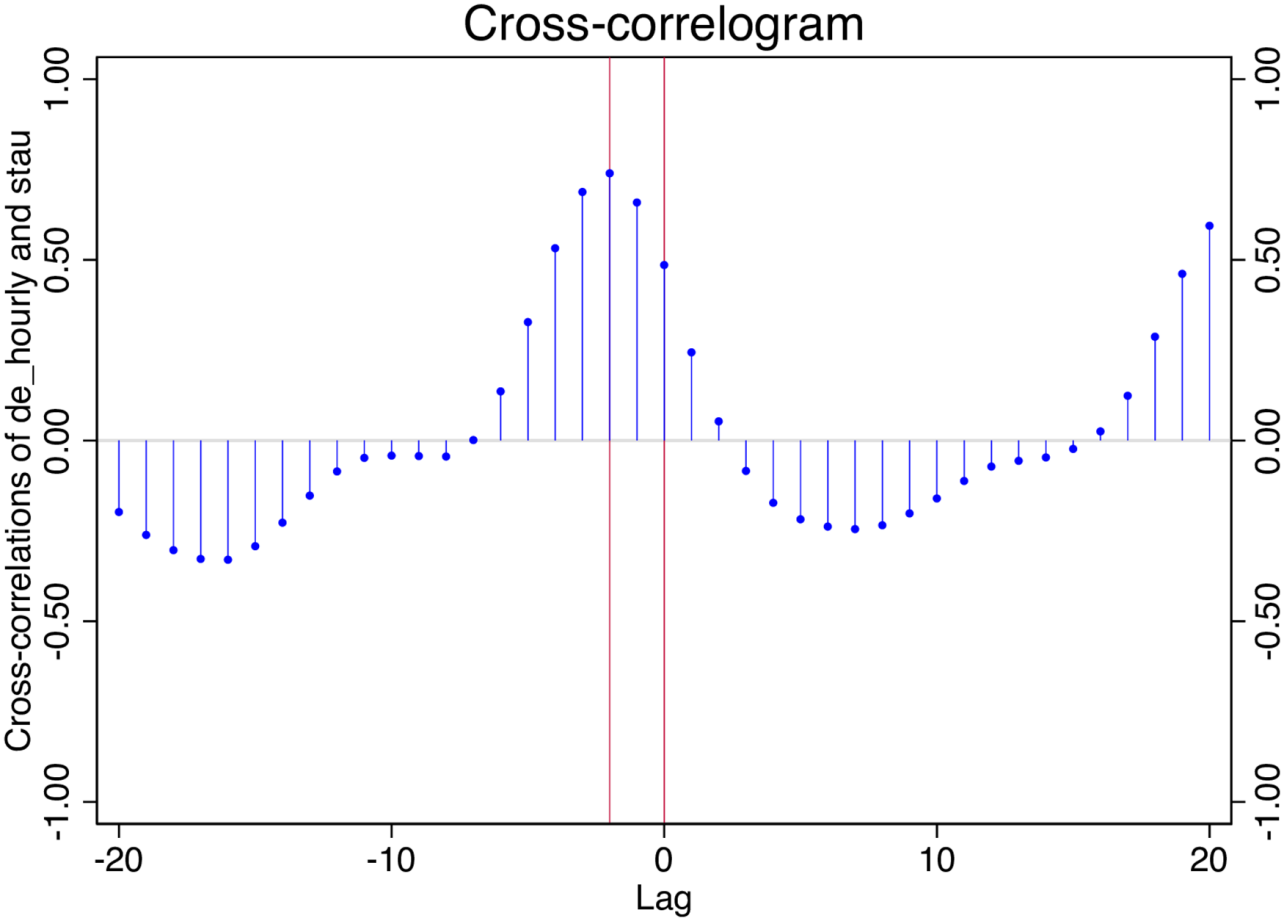
Google Searches for “stau” vs ADAC reports. A cross correlogram between the hourly number of ADAC traffic jam reports and the hourly Google search intensity for stau, based on the entire observation time interval of 51 days, establishes that Google search has a two hour advance on road conditions. Data Source: Google Trends, ADAC and own calculations.

Informed by the observation in Fig 4 I estimate five “Granger causality” ([14]) models:

$$S_{t}=\alpha S_{t-2}+\beta +\epsilon_{t}$$ (M1)

$$S_{t}=\alpha G_{t-2}+\beta +\epsilon_{t}$$ (M2)

$$S_{t}=\alpha D+\beta H+\gamma +\epsilon_{t}$$ (M3)

$$S_{t}=\alpha D+\beta H+\gamma S_{t-2}+\delta S_{t-24}+\epsilon +\epsilon_{t}$$ (M4)

$$S_{t}=\alpha D+\beta H+\gamma G_{t-2}+\delta +\epsilon_{t}$$ (M5)

where *S*_*t*_ is the natural log of the number of ADAC traffic jam reports at time t (hour), *D* is the day of the week (with values 0, 1, 2, 3, 4, 5 where 0 = Sunday), *H* is the hour of the day (with values 0−24) and *G*_*t*−2_ is the natural log of the Google search intensity for “stau” at time *t* − 2. We estimate the parameters *α*, *β*, *γ*, *δ*, *ϵ*; *ϵ*_*t*_ is the error term. I use partly overlapping letters for the intercepts in the models as there is no danger of confusion. The regressions are restricted to the fixed effects from days and hours that are statistically significant for at least one of the models.

Table 2 summarises the results of the five OLS regressions with each column being one model depicting its estimated intercepts and their statistical significance together with the model’s adjusted *R*^2^, Akaike Information Criterion and Root Mean Squared Error. The first two models M1 and M2 are the simplest possible models where we respectively regress the natural log of the number of ADAC reports on the (natural log of) its second lag and the (natural log of) the second lag of Google search intensity for stau, the model M3 explores the extend to which day-of-week and hour-of-day fixed effects suffice to explain the variation of the number of ADAC traffic jam reports. Model M4 is the full blown autoregressive model using fixed effects together with two hour and twenty-four hour lags. This model is the one which is used as the benchmark to beat and is meant to demonstrate the extend to which “historical” values of the series can predict it. Finally model M5 includes day-of-week and hour-of-day fixed effects together with the second lag of the Google stau searches. Clearly Eq M5 is the best model on all counts: lowest AIC, smallest RMSE and explains significantly more of the variation (85.1%) of our target variable than the second best model (67.3%). Since clearly Eq M2 beats Eqs M1 and M2 is the best model we have established our claim that the two hour lagged Google searches capture much of the stochastic behaviour of daily mobility which would otherwise remain elusive. Notice that the “historical” baseline model M4 ought to be similar to the prediction methods used by current systems using floating car data.

**Table 2:** Forecasting the number of ADAC traffic reports.

	M1	M2	M3	M4	M5
	*S*_*t*_	*S*_*t*_	*S*_*t*_	*S*_*t*_	*S*_*t*_
	coef./*p*-value	coef./*p*-value	coef./*p*-value	coef./*p*-value	coef./*p*-value
*S*_*t*−2_	.659***			.326***	
	(.000)			(.000)	
*S*_*t*−24_				.244***	
				(.000)	
*G*_*t*−2_		.368***			.415***
		(.000)			(.000)
D = 0			.000	.000	.000
			(.)	(.)	(.)
D = 1			.433***	.337***	.268***
			(.000)	(.000)	(.000)
D = 2			.465***	.240***	.337***
			(.000)	(.000)	(.000)
D = 3			.527***	.287***	.382***
			(.000)	(.000)	(.000)
D = 4			.559***	.301***	.399***
			(.000)	(.000)	(.000)
D = 5			.480***	.232***	.154***
			(.000)	(.000)	(.000)
D = 6			.076*	–.056	–.051*
			(.033)	(.110)	(.023)
H = 7			.000	.000	.000
			(.)	(.)	(.)
H = 8			.437***	.320***	.253***
			(.000)	(.000)	(.000)
H = 9			.565***	.345***	.342***
			(.000)	(.000)	(.000)
H = 10			.402***	.082	.304***
			(.000)	(.099)	(.000)
H = 11			.294***	–.037	.344***
			(.000)	(.479)	(.000)
H = 12			.293***	.016	.424***
			(.000)	(.739)	(.000)
H = 13			.323***	.075	.435***
			(.000)	(.114)	(.000)
H = 14			.383***	.121*	.423***
			(.000)	(.011)	(.000)
H = 15			.469***	.177***	.466***
			(.000)	(.000)	(.000)
H = 16			.562***	.230***	.492***
			(.000)	(.000)	(.000)
H = 17			.706***	.309***	.531***
			(.000)	(.000)	(.000)
H = 18			.778***	.332***	.552***
			(.000)	(.000)	(.000)
H = 19			.651***	.192***	.448***
			(.000)	(.001)	(.000)
const.	1.734***	4.631***	4.509***	1.974***	3.985***
	(.000)	(.000)	(.000)	(.000)	(.000)
Adj. *R*^2^	.434***	.631***	.612***	.693***	.851***
AIC	531.027	49.119	–57.311	–185.875	–639.008
RMSE	.306	.247	.227	.204	.141
No. of cases	1126.000	1126.000	611.000	598.000	609.000

*** *p* < 0.01,

** *p* < 0.05,

* *p* < 0.1

### Robustness Tests

My analysis has demonstrated that Google Search intensity for stau contains advance information on the number of traffic jams two hours before they occur. One can clearly not do much better than that since I only have a crude aggregate measure of road conditions. Better data would most likely allow better models to come to fruition. In this chapter I would like to perform some robustness tests to better support the validity of my results. I do so by offering scatter plots for data and their fit for the Google data model vs its nearest competitor models, by performing a rolling forecasting exercise and by performing a bootstrap regression to demonstrate that confidence intervals are robust.

**Fig 5:**
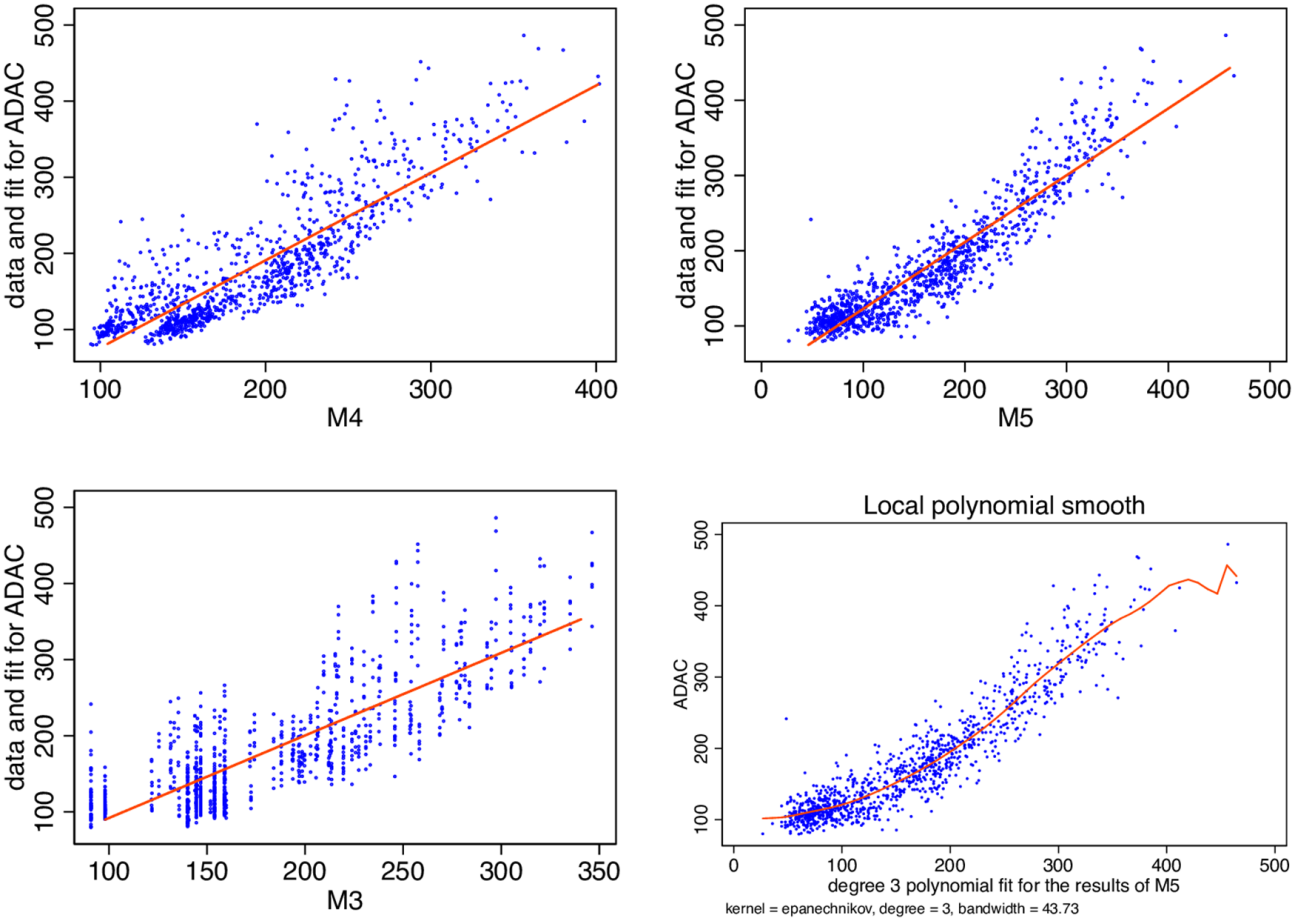
Comparing Eqs M3, M4 and M5. Clearly the regression line of Eq M5 captures the data better than that of Eqs M4 and M3. A higher degree term is apparent (bottom right). Data Source: Google Trends (www.google.com/trends), ADAC and own calculations.

Fig 5 contains scatter plots and regression lines for the naive fixed effects model M3, the historical model M4 and the mode M5 which is informed by the Google Trends data. Clearly Eq M5 fits the data best. It becomes apparent that higher degree terms are present in model M5. This is not surprising since the probability of pairwise interaction increases with the number of active drivers but reaches a saturation point after a certain critical value is reached. This is depicted in the bottom right of Fig 5 which shows a third degree polynomial fit.

A practitioner whose job is to analyse and predict road conditions can and will update his or her model as data becomes available. Since Google Trends data are updated hourly so can the prediction model using these data be continuously re-estimated. I performed a rolling forecast robustness test for models M4 and M5 by estimating the models up to 7 am for all but the last few days (for a total of 10 repetitions) and predicting the 9 am peak of the ADAC reports and similarly for the afternoon estimating up to 16:00hrs and predicting the 18:00hrs peak. I found that the RMSE in the morning trials is .36 for model M4 and .23 for model M5 whereas in the afternoon it is .22 and .17 respectively. In other words Eq M5 reduces the error by 36% in the morning and by 23% in the afternoon compared to the historical model.

Finally I used bootstrap regression to demonstrate the robustness of the confidence intervals when we forecast traffic jams with Google data. Since there is not enough data to do bootstrapping with day-of-week and hour-of-day effects I opted for a reduction of the data and two simpler models which on the one hand allow Google data to demonstrate their predictive power and on the other hand the bootstrapping technique to work. The first model regresses the daily number of ADAC report at 9:00 am on the Google search intensity for stau at 7:00 am. The second model similarly regresses the ADAC reports at 18:00 hrs on the Google search intensity for stau at 7:00 am and at 16:00 hrs. In both models we have a total of 51 data points. The models can be written as follows:

$$S_{d}^{9}=\alpha S_{d}^{7}+\beta +\epsilon_{d}$$ (*M_m_*)

$$S_{d}^{18}=\alpha S_{d}^{7}+\beta S_{D}^{16}+\gamma +\epsilon_{d},$$ (*M_a_*)

where *α*, *β*, *γ* are the intercepts to be estimated, *ϵ*_*d*_ is the error, *d* = 1, …, 51 counts the days in the dataset and $S_{d}^{x}$ for *x* = 7, 9, 16, 18 is the number of ADAC traffic jams at x hrs on day *d*.

For the morning model *M*_*m*_ we get *α* = .66 and *β* = 4. Their 95% confidence intervals are [.56, .76] and [3.82, 4.25] respectively and they become [.59, .72] and [3.89, 4.17] respectively after bootstrapping. The model explains 77% of the observed variation.

For the afternoon model *M*_*a*_ we estimate the intercepts to be *α* = .43, *β* = .28 and *γ* = 4.1. Their respective confidence intervals are [.31, .56], [.19, .38] and [3.8, 4.36] and they become [.32, .55], [.22, .35] and [3.85, 4.32] under bootstrapping. The model explains 72% of the observed variation.

## Discussion

Google search intensity for the word stau is an elegant and parsimonious way to capture driving intent in the near future in Germany and hence to predict road conditions two hours in advance by a method more informed than one which simply uses “historical data” or calendar fixed effects. Even after taking calendar fixed effects into account there is still a significant amount of stochasticity in the number of cars that drive off every day. Whether redeployed employees, private persons responding to unforeseen events or other stochastic changes there is a large amount of variation historical data cannot possibly capture. Google searches for stau are a good proxy for drivers telling us they will drive and are hence as close as we can currently come to having a public ledger of future traffic participants. I have demonstrated this by predicting the countrywide number of ADAC traffic jam reports, an admittedly crude proxy for road conditions. Better target data is needed and more research is necessary to operationalise the results of this paper which is hence only a proof of concept. Since Google searches for stau are often accompanied by city, region or highway information access to better data such as the floating car data of Google Traffic or other traffic jam information providers (coupled with geolocating the IP address from which the search is initiated) could allow one to build predictive systems and in fact even preventive systems. As a consequence Google Trends may predict Google Traffic and their combination might evolve into a prediction and prevention system. This paper is another indication that social science in the upcoming future will indeed look and feel more and more like “doing physics with particles that have feelings” (“*Imagine how much harder physics would be if electrons had feelings!*”—Richard Feynman, Caltech graduation ceremony).
